# The protective associations of breastfeeding with infant overweight and asthma are not dependent on maternal FUT2 secretor status

**DOI:** 10.3389/fnut.2023.1203552

**Published:** 2023-10-30

**Authors:** Melissa B. Manus, Stephanie K. Goguen, Meghan B. Azad

**Affiliations:** ^1^Manitoba Interdisciplinary Lactation Centre (MILC), Children’s Hospital Research Institute of Manitoba (CHRIM), Winnipeg, MB, Canada; ^2^Department of Pediatrics and Child Health, University of Manitoba, Winnipeg, MB, Canada

**Keywords:** infant feeding, human milk, maternal secretor status, infant asthma, infant weight gain

## Abstract

Breastfeeding supplies infant gut bacteria with human milk oligosaccharides (HMOs) as a nutrient source. HMO profiles are influenced by the FUT2 gene, which encodes an enzyme affecting the fucosylation of milk sugars. 20 to 40% of individuals have a “non-secretor” polymorphism that inactivates the FUT2 gene, resulting in variable HMO proportions in milk. This has engendered a concerning, yet unfounded, perception that non-secretor milk is “inferior.” To address this untested hypothesis, we re-analyzed two datasets in which we previously showed that breastfeeding was protective against early life asthma and excessive infant weight gain in the Canadian CHILD Cohort Study. Using stratified regression models, we found that the protective association of exclusive breastfeeding and infant asthma was not modified by maternal secretor status (secretors aOR: 0.53, 95% CI 0.31 to 0.92; non-secretors aOR: 0.36, 95% CI 0.12 to 1.04; *p* for interaction = 0.50, N = 2086 children). Similarly, the association of breastfeeding with lower infant BMI and weight gain velocity did not vary by maternal secretor status (infant BMI: secretors aβ −0.47, 95% CI −0.66 to −0.29; non-secretors aβ −0.46, 95% CI −0.78 to −0.13; *p* for interaction = 0.60; N = 1971 infants). Our results indicate that secretor and non-secretor mothers can equally promote infant growth and respiratory health through breastfeeding. These findings run contrary to the idea that non-secretor milk is an inferior food source, and instead reify the importance of breastfeeding for all infants. The results of this study can inform feeding recommendations that are applicable to all infants, regardless of maternal secretor status.

## Introduction

Breastfeeding provides myriad benefits to infants, including supplying infant gut bacteria with human milk oligosaccharides (HMOs) as a nutrient source (1). HMO profiles are strongly influenced by the FUT2 gene, which encodes an enzyme that affects the fucosylation of milk sugars (2). Globally, 20% to 40% of individuals (3) have a “non-secretor” polymorphism that inactivates the FUT2 gene. These individuals display altered enzymatic activity and a distinct milk profile that is enriched with certain HMOs, yet contains lower proportions of others (4). One HMO in particular, 2′-fucosyllactose (2’FL), is virtually absent in the milk of non-secretors (4). In contrast, 2’FL is typically the most abundant HMO in secretor milk.

Alongside growing interest in HMOs and their role in infant development, there is a concerning and largely unfounded perception that non-secretor milk is somehow “inferior (5).” In one study, the consumption of non-secretor milk was associated with delayed infant gut colonization by HMO-utilizing bacteria that are critical for immune system development (6), though other studies have failed to find associations between maternal secretor status and infant gut bacterial communities (7). Alongside variation in the gut microbiome based on maternal secretor status, evidence of a relationship between 2’FL exposure and infant cognitive development (8) has led to the speculation that non-secretor milk is “deficient.” However, the hypothesis that non-secretor milk is an inferior food source for infants has not been fully tested. These data are essential to informing public health messaging and research priorities related to maternal secretor status, infant feeding, and health. To address this gap, we re-analyzed two datasets in which we previously showed that breastfeeding was protective against early life asthma and excessive infant weight gain. The current analysis was motivated by the central question: *are the benefits of breastfeeding dependent on maternal secretor status?*

## Methods

### Study population

We leveraged data from two previously published studies that explored associations between infant feeding mode and asthma at 3 years (9), as well as BMI z score and weight gain velocity at 1 year (10) in the Canadian CHILD Cohort Study. This research was approved by the Human Research Ethics Boards at McMaster University and the Universities of Manitoba, Alberta, Toronto, and British Columbia.

### Secretor status, asthma diagnosis, and infant body mass index

Secretor status was defined using the methods in Moossavi et al. (11), for genotyping of the rs601338 and rs1047781 single nucleotide polymorphism in the *fucosyl-transferase* 2 (FUT2) gene. Asthma at 3 years of age was diagnosed (as possible or probable) by a healthcare professional, using medical history and physical examination (9). The diagnosis of “possible or probable asthma” reflects the fact that a firm asthma diagnosis is generally not possible until later in childhood. Body mass index was determined from weight and length measured by CHILD Study staff at the 12-month clinical assessment (10). Since measures of underweight, including stunting and wasting, are not common in this infant population (as evidence by the positive median z-scores), lower z-scores (i.e., closer to zero) in the current study indicate healthier body composition and weight gain trajectories. Weight gain velocity was calculated as the change in weight for age z-score during the period from birth to 12 months.

### Statistical analysis

We used base packages in R to clean and subset the data, allowing model stratification by maternal secretor status. The glm function within the *stats* package (12) was used to stratify regression models that included infant feeding mode as the exposure and either asthma diagnosis, BMI z score, or weight gain velocity as the outcome. We also formally tested for interactions between maternal secretor status and infant feeding mode for asthma at 3 years (logistic regression) and BMI z score and weight gain velocity at 1 year (linear regression). All models were adjusted for the same covariates used in the original studies (9, 10). Unadjusted model results are presented in Supplementary Figures S1, S2. All plots were generated using the *ggplot2* package (13). The figures display frequency data within the CHILD study, where participants represent a sample of the Canadian population. Since error bars help to visualize the (un)certainty in this estimate, they were calculated and included in the figures as a representation of the confidence intervals associated with sub-sampling from a larger population (9).

## Results

**Figure 1:**
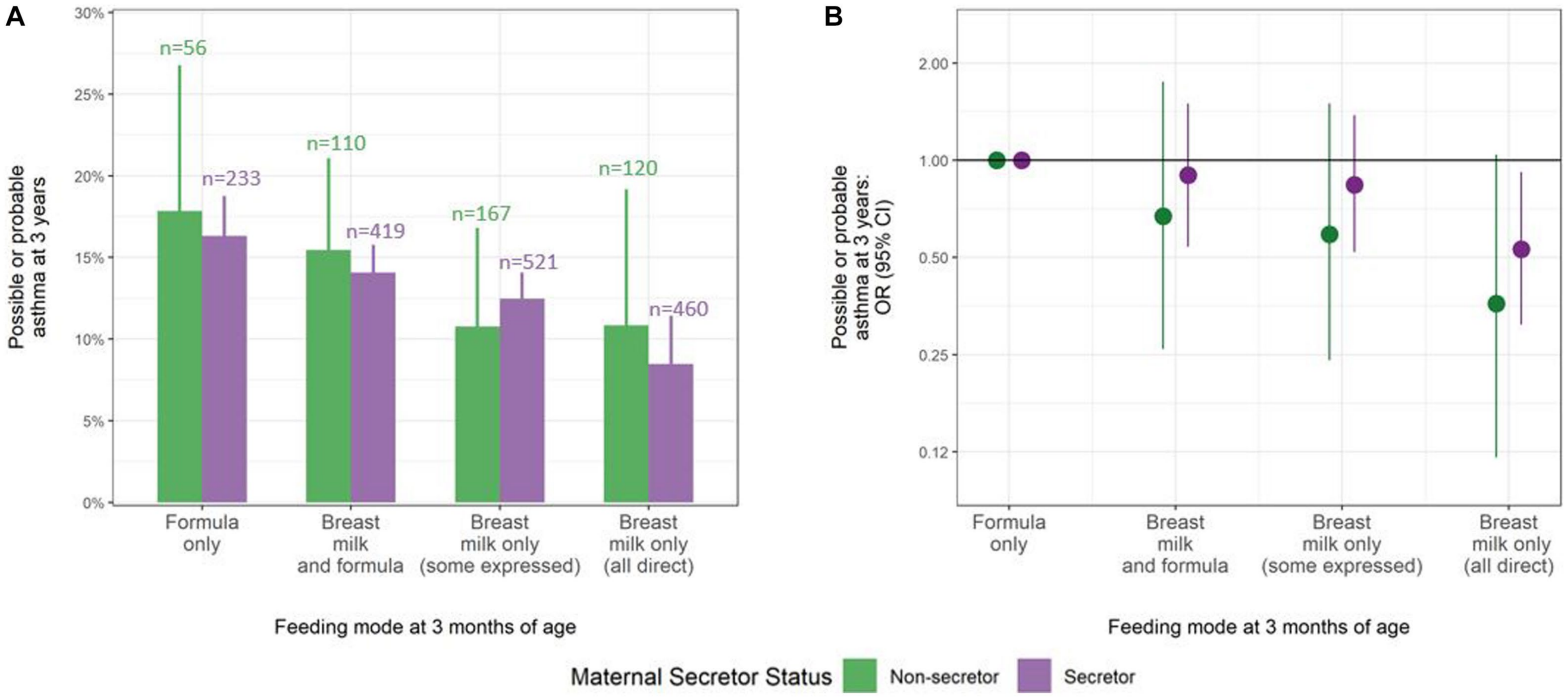
Consistent protective association between breastfeeding at 3 months and possible or probable asthma at 3 years, regardless of maternal secretor status. **(A)** Frequency of possible or probable asthma at 3 years by feeding mode at 3 months (total N = 2086). The values above the bars indicate the number of children in each feeding group, stratified by maternal secretor status. **(B)** Adjusted odds ratios (ORs) of possible or probable asthma at 3 years by feeding mode at 3 months. ORs adjusted for infant sex, maternal asthma, ethnicity, method of birth, daycare attendance, and gestational age, with multiple imputation of missing data. Lines represent 95% confidence intervals that display the (un)certainty in sub-sampling study participants from a larger population.

We found no evidence of an interaction between infant feeding mode and maternal secretor status for any of the health outcomes. The protective association of exclusive breastfeeding with asthma was not modified by maternal secretor status (*p* for interaction = 0.50; N = 2086 children) (Figure 1). Adjusting for covariates, infants exclusively breastfed by secretor mothers showed a 47% reduction in the risk of asthma at 3 years (aOR: 0.53, 95% CI 0.31 to 0.92) compared to infants who received only formula. Similarly, exclusively breastfed infants of non-secretor mothers displayed a 64% reduction in the risk of asthma at 3 years (aOR: 0.36, 95% CI 0.12 to 1.04). The unadjusted models produced similar results (secretor mothers: OR: 0.48, 95% CI 0.29 to 0.77; non-secretor mothers: OR: 0.56, 95% CI 0.23 to 1.40) (Supplementary Figure S1).

**Figure 2:**
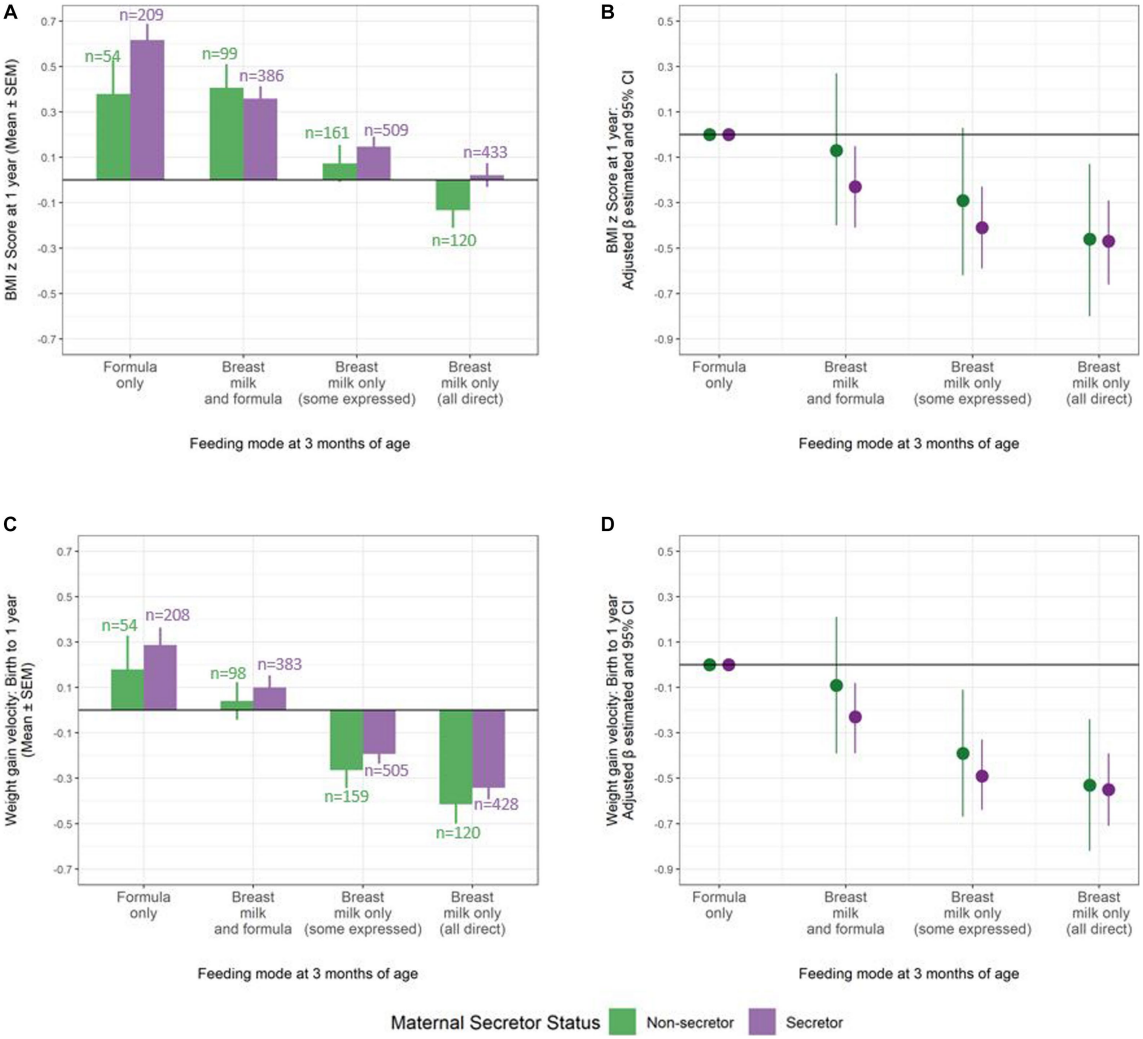
Consistent beneficial association of breastfeeding at 3 months with BMI z score and infant weight gain velocity at 1 year, regardless of maternal secretor status. **(A)** Mean BMI z score and **(B)** adjusted β estimates for BMI z score by infant feeding mode at 3 months (N = 1971 dyads). **(C)** Mean weight gain velocity and **(D)** adjusted β estimates for weight gain velocity by infant feeding mode at 3 months (N = 1955 dyads). Models adjusted for study site, maternal age, pre-pregnancy BMI, ethnicity, education, marital status, smoking during pregnancy, mode of delivery, parity, infant sex, gestational age, and birth weight. Lines represent 95% confidence intervals that display the (un)certainty in sub-sampling study participants from a larger population.

Similarly, the association of breastfeeding with healthier (lower) infant BMI and weight gain velocity did not vary by maternal secretor status (*p* for interaction = 0.60 and 0.81; N = 1971 and 1955 infants, respectively) (Figure 2). For example, in covariate-adjusted models, infants exclusively breastfed by a secretor mother had a 0.47 SD lower BMI z-score (aβ −0.47, 95% CI −0.66 to −0.29) compared to formula fed infants; virtually identical to the effect estimate for those exclusively breastfed by a non-secretor mother (aβ −0.46, 95% CI −0.78 to −0.13). The unadjusted models showed similar results (secretor mothers: β −0.59, 95% CI −0.77 to −0.42; non-secretor mothers: β −0.51, 95% CI −0.83 to −0.19) (Supplementary Figure S2).

## Discussion

We provide new evidence that the benefits of breastfeeding related to infant weight gain, body composition, and early childhood asthma are not dependent on maternal secretor status. Compared to receiving secretor milk, the consumption of non-secretor milk appears to provide a similar degree of dose-dependent protection against asthma and infant overweight. These data run contrary to the idea that non-secretor milk is an inferior food source for infants, and instead reify the importance of breastfeeding, regardless of maternal secretor status.

Further research should explore these associations in relation to other health outcomes and feeding scenarios. For example, data from the UK ALSPAC cohort suggest potentially enhanced protective effects of non-secretor milk against infant diarrhea (14). It will be particularly important to explore this relationship in contexts where infant mortality due to infectious disease remains high, as infections may provide selection pressure on the non-secretor polymorphism in these settings. Additionally, studies should further explore the connections between specific HMOs, the infant gut microbiome, and health outcomes in early life. Key bacterial colonizers, such as *Bifidobacterium*, may be able to utilize a range of HMOs beyond 2’FL (11). If so, then the benefits of breastfeeding that are likely mediated by the infant gut microbiome— including those explored in the current study (15, 16)— may extend to all breastfeeding infants, regardless of maternal secretor status and the associated abundance of 2’FL in milk.

Maternal secretor status may also be relevant to human donor milk, where the HMO profile of the pregnant person may not match the HMO profile of donor milk consumed postnatally. With evidence of fetal exposure to HMOs, including 2’FL, in amniotic fluid (17), infants may be primed to receive a specific amount or profile of HMOs *in utero*. While additional research is needed to understand the mechanisms and consequences of fetal exposure to HMOs, evidence of this phenomenon is in line with the Developmental Origins of Health and Disease literature, which posits that intrauterine exposure to bioactive molecules has a profound impact on birth outcomes and infant physiology (18). In the context of maternal secretor status, it may be that HMOs in amniotic fluid provide a “signal” of the composition of milk to which the infant will be exposed postnatally. Additional research on potential discordance between HMO exposure before and after birth is warranted. Future research should also consider the influence of *infant* secretor status on health outcomes (19), including the potential biological impacts of “mismatched” maternal–infant secretor status.

Overall, our current results indicate that secretor and non-secretor mothers can equally promote infant growth and protect against asthma through breastfeeding. These findings can inform feeding recommendations that are applicable to all infants, independent of maternal secretor status.

## Data availability statement

The data analyzed in this study is subject to the following licenses/restrictions: data will be made available upon request from the CHILD Cohort Study as described at https://childstudy.ca/for-researchers/data-access/. Requests to access these datasets should be directed to child@mcmaster.ca.

## Ethics statement

The studies involving humans were approved by The Human Research Ethics Boards at McMaster University and the Universities of Manitoba, Alberta, Toronto, and British Columbia. The studies were conducted in accordance with the local legislation and institutional requirements. Written informed consent for participation in this study was provided by the participants’ legal guardians/next of kin.

## Author contributions

MA conceptualized and designed the study, and critically reviewed and revised the manuscript. MM drafted the initial manuscript and critically reviewed and revised the manuscript. SG carried out the initial analyses and critically reviewed and revised the manuscript. All authors contributed to the article and approved the submitted version.
