# Supplementary material for: The protective associations of breastfeeding with infant overweight and asthma are not dependent on maternal FUT2 secretor status
Source: Front Nutr. 2023 Oct 30;10:1203552. doi: 10.3389/fnut.2023.1203552 (PMC10642293; doi:10.3389/fnut.2023.1203552)
Supplement: Supplementary file 2 [file Data_Sheet_2.DOCX]

**
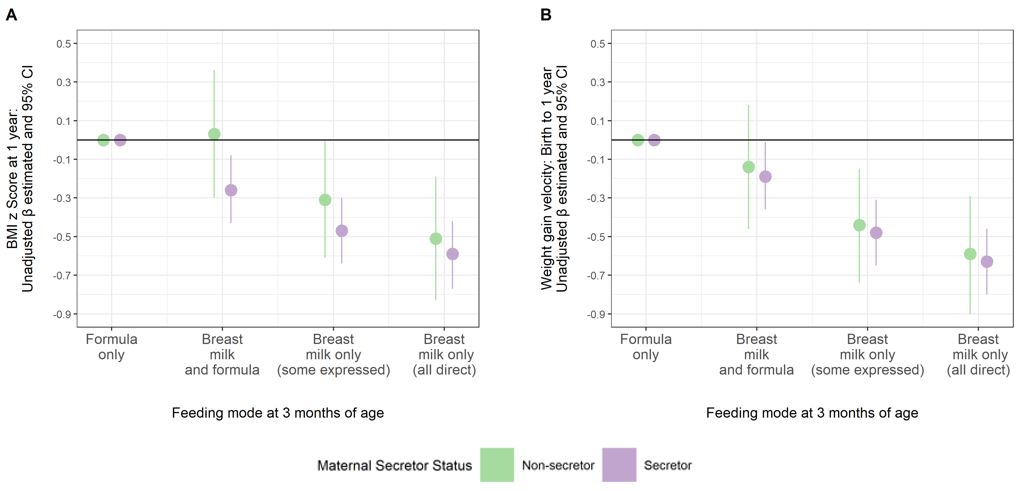
**

**Figure S2.** Adjusted associations from multivariable regression models are shown in Figure S2. Unadjusted models display a consistent beneficial association of breastfeeding at 3 months with BMI z score and infant weight gain velocity at 1 year, regardless of maternal secretor status. Unadjusted β estimates for **(A)** BMI z score and **(B)** weight gain velocity by infant feeding mode at 3 months. Lines represent 95% confidence intervals that display the (un)certainty in sub-sampling study participants from a larger population.
